# Impact of COVID-19 pandemic on the incidence of otitis media with effusion in adults and children: a multicenter study

**DOI:** 10.1007/s00405-021-06958-4

**Published:** 2021-07-04

**Authors:** Giannicola Iannella, Giuseppe Magliulo, Jerome R. Lechien, Antonino Maniaci, Tiziano Perrone, Pier Carlo Frasconi, Andrea De Vito, Chiara Martone, Salvatore Ferlito, Salvatore Cocuzza, Giovanni Cammaroto, Giuseppe Meccariello, Valentina Monticone, Antonio Greco, Marco de Vincentiis, Massimo Ralli, Vincenzo Savastano, Serena Bertin, Annalisa Pace, Alessandro Milani, Roberta Polimeni, Stefano Pelucchi, Andrea Ciorba, Claudio Vicini

**Affiliations:** 1 Otolaryngology, Head-Neck and Oral Surgery Unit, Department of Head-Neck Surgery, Morgagni Pierantoni Hospital, Via Carlo Forlanini, 34, 47121 Forli, Italy; 2 Department of ‘Organi di Senso’, University “Sapienza”, Viale dell’Università, 33, 00185 Rome, Italy; 3 Laboratory of Anatomy and Cell Biology, Faculty of Medicine, University of Mons (UMONS), Avenue du Champ de mars, 6, 7000 Mons, Belgium; 4 Department of Medical and Surgical Sciences and Advanced Technologies “GF Ingrassia”, ENT Section, University of Catania, Via S. Sofia, 78, 95125 Catania, Italy; 5 Department ENT and Audiology, University of Ferrara, Via Savonarola, 9, 44121 Ferrara, Italy; 6 Otolaryngology, Head-Neck and Oral Surgery Unit, Department of Head-Neck Surgery, Ospedale “Santa Maria Delle Croci”, Viale Vincenzo Randi, 5, 48121 Ravenna, Italy; 7 Otolaryngology Department, Azienda Sanitaria Locale CN2, Alba, Italy

**Keywords:** COVID-19, Otitis media effusion, OME, Eustachian tube dysfunction, Upper airways respiratory infections

## Abstract

**Purpose:**

To compare and analyze the incidence of otitis media with effusion (OME), before and during the COVID-19-related pandemic period, to evaluate the effects of the social changes (lockdown, continuous use of facial masks, social distancing, reduction of social activities) in the OME incidence in children and adults.

**Methods:**

The number of diagnosed OME in e five referral centers, between 1 March 2018 and 1 March 2021, has been reviewed and collected. To estimate the reduction of OME incidence in children and adults during the COVID-19 pandemic period the OME incidence in three period of time were evaluated and compared: group 1—patients with OME diagnosis achieved between 1/03/2018 and 01/03/2019 (not pandemic period). Group 2—patients with OME diagnosis achieved between 1/03/2019 and 1/03/2020 (not pandemic period). Group 3—patients with OME diagnosis achieved between 1/03/2020 and 1/03/2021 (COVID-19 pandemic period).

**Results:**

In the non-pandemic periods (group 1 and 2), the incidence of OME in the five referral centers considered was similar, with 482 and 555 diagnosed cases, respectively. In contrast, the OME incidence in the same centers, during the pandemic period (group 3) was clearly reduced with a lower total number of 177 cases of OME estimated. Percentage variation in OME incidence between the first non-pandemic year considered (group 1) and the pandemic period (group 3) was—63, 3%, with an absolute value decrease value of—305 cases. Similarly, comparing the second non-pandemic year (group 2) and the pandemic year (group 3) the percentage variation of OME incidence was—68, 1% with an absolute value of—305 cases decreased.

**Conclusions:**

Our findings showed a lower incidence of OME during the pandemic period compared with 2 previous non pandemic years. The drastic restrictive anti-contagion measures taken by the Italian government to contain the spread of COVID-19 could have had a positive impact on the lower OME incidence during the last pandemic year.

## Introduction

Otitis media with effusion (OME) is defined as the presence of non-infected fluid in the middle ear cavity, without signs or symptoms of an acute ear infection. This condition is characterized by conductive hearing loss, ear fullness and occasionally by pain from pressure difference between the nasopharynx and middle ear [1, 2].

The most accredited pathogenetic theories consider OME as a consequence of a viral or bacterial upper respiratory tract infection (URTI) that causes an Eustachian tube dysfunction (ETD) and pressure changes in the middle ear. Middle ear negative pressure is associated with an inflammatory immune reaction against rhino-pharyngeal infections, which could lead to the increase in number and activity of middle ear muciparous cells with the effusion generation [1–7].

Since the end of February 2020, the Coronavirus (COVID-19) has created a worldwide deadly pandemic that has become the major public health problem in world. Social distancing, long periods of lockdown and facial masks, have been carried out in different countries around the world to limit the infection spread [8–12].

Social distancing and the use of personal protective equipment (PPE) have been proven to be effective in reducing the SARS-COV-2 transmission. Different authors have shown as these measures have also reduced the upper airways colonization by other pathogens (viruses, bacteria and irritating substances), with a significant decrease in acute respiratory infections in children and adults [13]. Moreover, recent winter surveillance data from Australia, Korea and Japan have demonstrated a decrease in seasonal influenza activity compared to previous seasons [4–6]. Hence, it is plausible that public health interventions COVID-19 related are having a beneficial impact on the prevention of other respiratory pathogens infection [13–16].

Is therefore possible that social changes imposed by the COVID-19 pandemic have also modified the incidence of effusive otitis media in children and adults due to a reduction of upper respiratory infections?

The aim of the present study has been to compare and analyze the incidence of OME, before and during the COVID-19-related pandemic period, to evaluate the effects of the social changes (lockdown, continuous use of facial masks, social distancing, and reduction of social activities) in the OME incidence in children and adults.

To our knowledge, the present study represents the first report that analyzes the effects of 1-year social restrictions on OME incidence.

## Materials and methods

This is a multicentric retrospective study performed in five Otolaryngology Departments of tertiary referral centers in Italy: Morgagni Pierantoni Hospital of Forlì, Policlinico Umberto I Hospital -Sapienza University of Rome, Santa Maria delle Croci Hospital of Ravenna, Policlinico Hospital—University of Catania, Hospital of ‘Infermi’ Faenza.

### Study design

The number of diagnosed OME, in the five referral centers, between 1 March 2018 and 1 March 2021 were initially reviewed from the medical charts and collected. This time frame included the new diagnoses of OME made in the 2 years before the COVID-19 spread and the ones made in the year of the COVID-19 pandemic.

To estimate the reduction of OME incidence in children and adults during the COVID-19 pandemic period all patients initially enrolled were divided into three groups according to the following time span:

- Group 1—patients with OME diagnosis achieved between 1/03/2018 and 01/03/2019 (not pandemic period).
- Group 2—patients with OME diagnosis achieved between 1/03/2019 and 1/03/2020 (not pandemic period).
- Group 3—patients with OME diagnosis achieved between 1/03/2020 and 1/03/2021 (COVID-19 pandemic period).

Two subgroups within the pandemic period were also considered: one between 01/03/2020 and 30/05/2020, corresponding to the national lockdown period in Italy, and a second period between 1/06/2020 and 01/03/2021, corresponding to the pandemic period characterized by severe national restrictions, without a national lockdown. In both of these periods, the use of the facial masks and social distancing was mandatory in Italy.

### Inclusion and exclusion criteria

In all centers OME diagnosis were performed, according to the commonly recognized OME diagnostic criteria, if all the following criteria were fulfilled:

- Type B (flat) tympanogram;
- Reported hearing loss and/or ear fullness with otomicroscopic evidence of middle ear effusion, defined by a yellowish retracted tympanic membrane and by air-fluid level or bubbles in the middle ear;
- Mild to moderate conductive hearing loss diagnosed by pure tone audiometry.

Tympanograms were obtained with a standard 226 Hz probe tone and classified as: type A: normal compliance and middle ear pressure. Type B: low compliance with no discernible peak. Type C: normal compliance with negative middle ear pressure, often associated with a retracted tympanic membrane due to Eustachian tube dysfunction.

Air-conduction (AC) and bone-conduction pure tone audiometry or behavioral audiometry with AC and BC auditory brainstem responses were used to assess the typical air-bone gap of conductive hearing loss.

Exclusion criteria for study enrollment were as following:

- Age < 6 months;
- Otomicroscopic evidence of tympanosclerosis, cholesteatoma, eardrum perforation, or complete stenosis or atresia of the external auditory canal;
- Craniofacial anomalies, cleft palate, or syndromes characterized by anatomic and functional impairment of the Eustachian tube;
- Contraindications to tympanometry, such as otitis externa, otorrhea, recent ear surgery (e.g., myringoplasty, tympanoplasty, and stapedectomy), presence of tympanostomy tubes, foreign body in the external auditory canal;
- Uncertain diagnosis of otitis media with effusion;
- Patients with a previous diagnosis of effusive otitis media were excluded from the study to avoid a possible bias;
- Patients lost to follow-up.

OME healing was also extrapolated from medical charts. The criteria to define OME resolution were as follows: change from type B to type A tympanogram; recovery of symptoms and conductive hearing loss; normal aspect of the eardrum on otomicroscopy.

Healing patients were sub-divided into those who recovered medically and those who were unresponsive to medical therapy and who had improved after placement of a ventilation tube.

### Statistical analysis and ethical statement

This research study was made in accordance with the principles of the Declaration of Helsinki. Given the retrospective nature of the study and anonymity, Ethic Committee approval was not required. To test the differences among groups, a chi-square test was used for analyzing categorical data differences. A demographic statistic of patient’s groups was performed. The percent variance of OME incidence between the different time periods was calculated.

## Results

Incidence of OME diagnosed in the three different time periods considered (group 1, 2 and 3) is shown in Table 1. In the non-pandemic periods (group 1 and 2), the incidence of OME in the 5 referral centers considered was similar, with 482 and 555 diagnosed cases, respectively. In contrast, the OME incidence in the same centers, during the pandemic period (group 3) was clearly reduced with a lower total number of 177 cases of OME estimated.

**Table 1:** Otitis media with effusion diagnosed in the three different periods of time in children and adults considered in the study

Time range	Total diagnosticate OME	Adults number/percentage	Children number/percentage
Group 1 01/03/2018–01/03/2019	482	189 (39, 2%)	293 (60, 8%)
Group 2 01/03/2019–01/03/2020	555	234 (42, 2%)	321 (57, 8%)
Group 3 01/03/2020–01/03/2021	177	103 58, 2%)	74 (41, 8%)
01/03/20–30/05/20 National lockdown	35	22 (62, 9%)	13 (37, 1%)
01/06/20–01/02/21	142	81 (57, 0%)	61 (43, 0%)

Percentage variation in OME incidence between the first non-pandemic year considered (group 1) and the pandemic period (group 3) was—63, 3%, with an absolute value decrease value of—305 cases. Similarly, comparing the second non-pandemic year (group 2) and the pandemic year (group 3) the percentage variation of OME incidence was—68, 1% with an absolute value of—305 cases decreased.

**Fig. 1:**
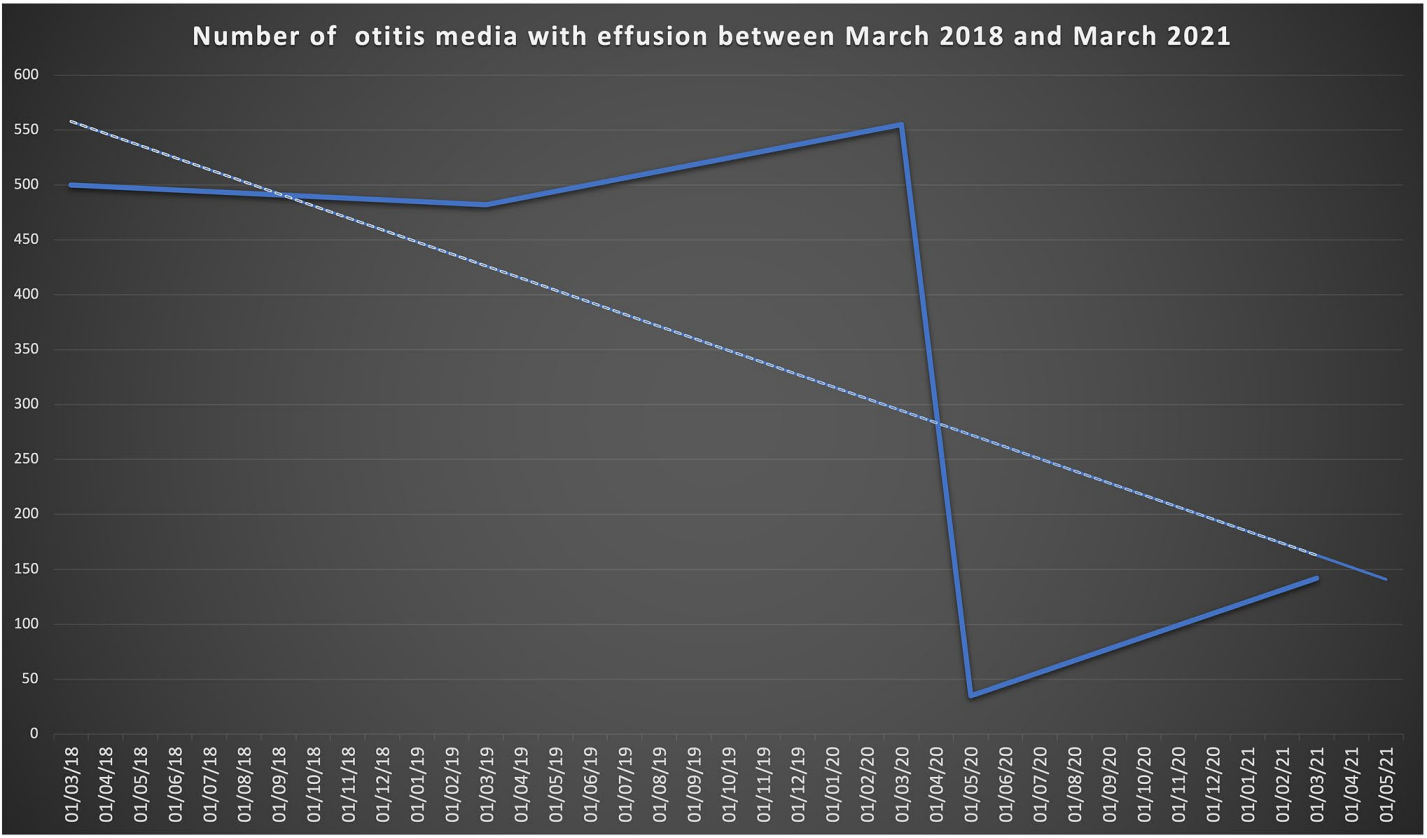
Otitis media with effusion diagnosticated between March 2018 and March 2021 showing the reducing trend over time

Figure 1 shows the graph of OME cases with a reducing trend over time.

In the first non-pandemic period (group 1), the OME diagnosis were performed in 39, 2% of adults and 60.8% of children. Similarly, in the second non-pandemic period (group 2), to 42, 2% of adults and 57, 8% were diagnosed the OME (Table 1). About the incidence of OME in adult and children, no statistical difference emerged between these two non-pandemic periods (*p* = 0.3). On the other hand, in the pandemic period, the OME diagnosis were made in higher percentage of adults (58.2%) compared with children (41.8%). A statistical difference emerged comparing group 1 and 2 versus group 3 (pandemic period) regarding incidence of OME in children and adults (*p* < 0.05 in each groups comparison; Table 1).

The percentages of affected sides, bilateral forms, gender and the average age of adults and children affected by OME in the three groups considered is shown in Table 2. No statistical difference was found in the patient’s characteristics (age and sex) and in the mono and bilateral forms between the three groups (*p* > 0.05 for each group comparison).

**Table 2:** Characteristics of patients with OME diagnosis in the three different periods of time considered in the study

	Group 1 01/03/2018–01/03/2019	Group 2 01/03/2019–01/03/2020	Group 3 01/03/2020–01/03/2021	***p*** chi square test	
				Group 1 vs group 3	Group 2 vs group 3
Female	233	270	96	0.1	0.2
Male	249	285	81		
Left side number/percentage	205 (42, 5%)	234 (42, 2%)	74 (41, 8%)	0.2	0.3
Right side number/percentage	201 (41, 7%)	201 (41, 7%)	201 (41, 7%)		
Bilateral OME number/percentage	76 (15, 8%)	90 (16, 2%)	26 (14, 7%)		
Average age (children)	6.4 ± 2.8	5.5 ± 3.6	4.9 ± 4.1	0.6 (*t* student test)	0.7 (*t* student test)
Average age (adults)	46.4 ± 22.3	52.5 ± 21.6	44.9 ± 24.6	0.8 (*t* student test)	0.7 (*t* student test)
Resolution with medical therapy	426 (88, 4%)	473 (85, 2%)	147 (83, 1%)	0.1	0.4
Ventilation tube placement	56 (11, 6%)	82 (14, 8%)	30 (16, 9%)		

Patients healed with medical therapy and patients treated with the positioning of a ventilation tube, in the three different time periods considered, are shown in Table 2. It did not emerge a statistical difference of the number of patients treated with medical therapy and those who underwent a trans-tympanic ventilation tube insertion between these periods (> 0.05 for each group comparison).

## Discussion

In 2020, the COVID-19 has been declared a Public Health Emergency of international concern by the World Health Organization (WHO). To reduce the number of SARS-CoV-2 infections, different countries have adopted restrictive anti-contagion measures (lockdown, continuous use of facial masks, social distancing, and reduction of social activities) [10–12, 17].

A nationwide lockdown was imposed by the Italian government from March 9 to May 18, 2020, to contain the COVID-19 spread. All schools and day care centers were completely closed, and people were strictly forbidden to leave their homes. Differently, from June 2020 until April 2021 were applied restrictive measures to avoid social contacts (such as closing restaurants, schools, shops, public meeting places, etc) in according to the COVID-19 infection rate. In addition, in this period all people were required to wear a face covering outdoors and/or in public places [18–20]. Facial masks have been proven to be an effective method of protection in this pandemic, which both reduces the exhalation of virus-laden aerosols from a COVID patient and minimizes the inhalation of airborne virus-laden aerosols by the subjects surrounding the patient [12, 17, 21]. This social scenario of restrictive measures adopted by the authorities represent a unique and exceptional event in recent world history and provided a great opportunity to evaluate the impact of social isolation to upper respiratory infections.

Different studies to date have shown as obligated avoidance of interpersonal contacts, rigorous respect of the hygienic-behavioral rules, and facial masks have reduced the spread of not only of SARS-CoV-2, but also other viruses and bacteria that underlie the development of common upper respiratory diseases [12–20].

Data of Monika Redlberger-Fritz et al. [15] show that national lockdown in Austria was associated to a significant impact on the prevalence of different respiratory viruses. Infection of influenza virus, respiratory syncytial virus, human metapneumovirus and rhinovirus cases were analyzed and compared between the season 2019/2020 and the five previous seasons. Authors observed a rapid and statistically significant reduction of cumulative cases for all these viruses within short time after the lockdown in March 2020, compared to previous seasons (*p* < 0.001).

Recently, an Italian study described that lockdown measures lead to reduction effects onto the epidemic of chickenpox, rubella, pertussis or measles, showing that a lockdown may reduce the prevalence of different infectious diseases [16].

The aim of the present study has been to compare the incidence of OME, before and during the COVID-19-related pandemic period, to evaluate the effects of the social changes in the OME incidence in children and adults.

A preliminary study about this topic has been published by Aldè et al. that has evaluated the effect of the Italian lockdown in modifying the OME incidence in children [20]. Authors have assessed the incidence of OME among children aged 6 months to 12 years who attended to the otologic division for hearing or vestibular disorders during 2 periods before the lockdown, May–June 2019 (*n* = 350) and January–February 2020 (*n* = 366), and the period immediately after the lockdown, May–June 2020 (*n* = 216).

The incidence of OME in this clinic population was 40.6% in May–June 2019, 52.2% in January–February 2020, while it was drastically reduced at 2.3% in May–June 2020. Children with chronic OME had a higher rate of disease resolution in May–June 2020 (93.3%) than those examined in May–June 2019 (20.7%, *p* < 0.001). According to these evidences, authors concluded that closure of schools and the physical distancing rules were correlated with a reduction in the incidence of OME after lockdown period [20].

In this multicenter study, we have evaluated OME incidence in children and adults in a longer pandemic period (1 years). We have estimated the OME incidence in the 2 years preceding the COVID-19 pandemic and used this value as a measure of comparison to show if there was a reduction in OME incidence during the pandemic year.

In the first (1/03/2018–01/03/2019) and second (1/03/2019–01/03/2020), non-pandemic period analyzed the OME incidence was similar with 482 and 555 diagnosed cases, respectively. On the other hand, the OME incidence during the pandemic period evaluated (1/03/2020–1/03/2021) was lower than of the previous 2 years with a total number of 177 cases of OME calculated. Percentage variation in OME incidence between the pandemic period and previous years considered was estimated to be—63, 3% and—68, 1%, respectively.

Evaluations of the 3-month period of strict lockdown demonstrated a very low incidence of OME with only 35 cases (62, 9% adults and 37.1% children) diagnosed in the 5 centers.

How could this drastic reduction in OME incidence during the pandemic be explained?

In our opinion, this event could be related to the main pathogenetic mechanism OME-related [1, 2]. This pathology is thought to be initiated by inflammatory and immune reactions against a rhino-pharyngeal infection. Viruses, including respiratory syncytial virus, rhinovirus, adenovirus, bocavirus, influenza virus, parainfluenza virus, enterovirus and human metapneumovirus, are known causes of upper respiratory infections and acute otitis media. These viruses can be the sole infective cause of acute otitis media or play a role in coinfection with bacteria. Upper respiratory infections induce a nasopharyngeal inflammation which lead to an Eustachian tube dysfunction/occlusion and a following negative middle-ear pressure [22–26]. Negative middle-ear pressure predisposes the middle ear to effusion formation, due to alterations in endotympanic gas exchange and in the secretory properties of the glands of the tympanic and mastoid submucosa. Considering this cascade of events, it is probable that the elimination of nasopharyngeal infections (‘primum movens’ of OME), obtained with social distancing and facial masks, has consequently led to a reduction in effusive otitis [1–4, 22–24].

During the pandemic period, the OME diagnosis were made in higher percentage of adults (58.2%) compared with children (41.8%) with an inverse trend compared to the previous years. A statistical difference emerged comparing group 1 and 2 with group 3 (pandemic period) regarding incidence of OME in children and adults (*p* < 0.05 in each groups comparison). In our opinion this data, which shows a greater reduction of OME in children, could be explained by the greater social changes imposed to the pediatric population during the last year. In all the pandemic period, schools have been almost always closed and children did distance learning. Differently, fewer limitations were imposed to workplaces for adults that resulted more exposed to social contact [18, 19].

Despite the interesting evidence shown on the incidence of OME during the last year of the pandemic, the study has several limitations. First, it is retrospective and despite this being a multicenter study a limited number of patients were considered.

In addition, there are a few sources of bias, including the possibility that some patients postponed or sought treatment elsewhere, as well as a reasonable reluctance of patients to come to the hospital for a follow-up visit due to the COVID-19 outbreak. A further prospective study of more patients is underway to validate these preliminary results.

## Conclusion

Our findings showed a lower incidence of OME during the pandemic period compared with 2 previous non pandemic years. The drastic restrictive anti-contagion measures taken by the Italian government to contain the spread of COVID-19 could have had a positive impact on the lower OME incidence during the last pandemic year.

## References

[1] Vanneste P, Page C (2019). Otitis media with effusion in children: pathophysiology, diagnosis, and treatment. A review. J Otol.

[2] Mills R, Hathorn I (2016). Aetiology and pathology of otitis media with effusion in adult life. J Laryngol Otol.

[3] Manno A, Iannella G, Savastano V, Vittori T, Bertin S, Pasquariello B, Pace A, Rossetti V, Magliulo G (2021) Eustachian tube dysfunction in children with adenoid hypertrophy: the role of adenoidectomy for improving ear ventilation. Ear Nose Throat J. 10.1177/0145561321989455 [Epub ahead of print] 10.1177/0145561321989455 33470833

[4] Homøe P, Heidemann CH, Damoiseaux RA, Lailach S, Lieu JEC, Phillips JS, Venekamp RP (2020). Panel 5: impact of otitis media on quality of life and development. Int J Pediatr Otorhinolaryngol.

[5] Juszczak HM, Loftus PA (2020). Role of allergy in Eustachian tube dysfunction. Curr Allergy Asthma Rep.

[6] Torretta S, Drago L, Marchisio P, Ibba T, Pignataro L (2019). Role of biofilms in children with chronic adenoiditis and middle ear disease. J Clin Med.

[7] Iannella G, Lucertini M, Pasquariello B, Manno A, Angeletti D, Re M, Magliulo G (2017). Eustachian tube evaluation in aviators. Eur Arch Otorhinolaryngol.

[8] Sharma A, Tiwari S, Deb MK, Marty JL (2020). Severe acute respiratory syndrome coronavirus-2 (SARS-CoV-2): a global pandemic and treatment strategies. Int J Antimicrob Agents.

[9] Maniaci A, Iannella G, Vicini C, Pavone P, Nunnari G, Falsaperla R, Di Mauro P, Ferlito S, Cocuzza S (2020). A case of COVID-19 with late-onset rash and transient loss of taste and smell in a 15-year-old boy. Am J Case Rep.

[10] Novelli G, Biancolella M, Mehrian-Shai R, Colona VL, Brito AF, Grubaugh ND, Vasiliou V, Luzzatto L, Reichardt JKV (2021). COVID-19 one year into the pandemic: from genetics and genomics to therapy, vaccination, and policy. Hum Genomics.

[11] Birimoglu Okuyan C, Begen MA (2021) Working from home during the COVID-19 pandemic, its effects on health, and recommendations: the pandemic and beyond. Perspect Psychiatr Care. 10.1111/ppc.12847 [Epub ahead of print] 10.1111/ppc.12847 PMC8242705 34003489

[12] Vicini C, Cammaroto G, Meccariello G (2020). Overview of different modified full-face snorkelling masks for intraoperative protection. Acta Otorhinolaryngol Ital.

[13] Kuitunen I, Artama M, Mäkelä L, Backman K, Heiskanen-Kosma T, Renko M (2020). Effect of social distancing due to the COVID-19 pandemic on the incidence of viral respiratory tract infections in children in Finland during early 2020. Pediatr Infect Dis J.

[14] Oster Y, Michael-Gayego A, Rivkin M, Levinson L, Wolf DG, Nir-Paz R (2020). Decreased prevalence rate of respiratory pathogens in hospitalized patients during the COVID-19 pandemic: possible role for public health containment measures?. Clin Microbiol Infect.

[15] Redlberger-Fritz M, Kundi M, Aberle SW, Puchhammer-Stöckl E (2021). Significant impact of nationwide SARS-CoV-2 lockdown measures on the circulation of other respiratory virus infections in Austria. J Clin Virol.

[16] Belingheri M, Paladino ME, Piacenti S, Riva MA (2021). Effects of COVID-19 lockdown on epidemic diseases of childhood. J Med Virol.

[17] Maniaci A, Ferlito S, Bubbico L, Ledda C, Rapisarda V, Iannella G, La Mantia I, Grillo C, Vicini C, Privitera E, Coco S, Cammaroto G, Lechien JR, Magliulo G, Pace A, Meccariello G, Cocuzza S (2021) Comfort rules for face masks among healthcare workers during COVID-19 spread. Ann Ig. 10.7416/ai.2021.2439 [Epub ahead of print] 10.7416/ai.2021.2439 33797548

[18] Pierri MD, Alfonsi J, Cefarelli M, Berretta P, Di Eusanio M (2021). COVID 19—perspective of an Italian center. J Card Surg.

[19] Caristia S, Ferranti M, Skrami E, Raffetti E, Pierannunzio D, Palladino R, Carle F, Saracci R, Badaloni C, Barone-Adesi F, Belleudi V, Ancona C, AIE working group on the evaluation of the effectiveness of lockdowns (2020). Effect of national and local lockdowns on the control of COVID-19 pandemic: a rapid review. Epidemiol Prev.

[20] Aldè M, Di Berardino F, Marchisio P, Cantarella G, Ambrosetti U, Consonni D, Zanetti D (2021). Effects of COVID-19 Lockdown on otitis media with effusion in children: future therapeutic implications. Otolaryngol Head Neck Surg.

[21] Liao M, Liu H, Wang X, Hu X, Huang Y, Liu X, Brenan K, Mecha J, Nirmalan M, Lu JR (2021). A technical review of face mask wearing in preventing respiratory COVID-19 transmission. Curr Opin Colloid Interface Sci.

[22] Angeletti D, Pace A, Iannella G, Rossetti V, Colizza A, Visconti IC, Gulotta G, Messineo D, de Vincentiis M, Greco A, D'Ambrosio F, Magliulo G (2021). Chronic obstructive Eustachian tube dysfunction: CT assessment with Valsalva maneuver and ETS-7 score. PLoS ONE.

[23] Hamilos DL (2019). Biofilm formations in pediatric respiratory tract infection : part 1: biofilm structure, role of innate immunity in protection against and response to biofilm, methods of biofilm detection, pediatric respiratory tract diseases associated with mucosal biofilm formation. Curr Infect Dis Rep.

[24] Quaranta N, Iannuzzi L, Gelardi M (2014). Does the type of rhinitis influence development of otitis media with effusion in children?. Curr Allergy Asthma Rep.

[25] Schilder AG, Marom T, Bhutta MF, Casselbrant ML, Coates H, Gisselsson-Solén M, Hall AJ, Marchisio P, Ruohola A, Venekamp RP, Mandel EM (2017). Panel 7: otitis media: treatment and complications. Otolaryngol Head Neck Surg.

[26] Cocuzza S, Maniaci A, Di Luca M, La Mantia I, Grillo C, Spinato G, Motta G, Testa D, Ferlito S (2020). Long-term results of nasal surgery: comparison of mini-invasive turbinoplasty. J Biol Regul Homeost Agents.

